# APOE as potential biomarkers of moyamoya disease

**DOI:** 10.3389/fneur.2023.1156894

**Published:** 2023-05-09

**Authors:** Haibin Wu, Jiang Xu, Jiarong Sun, Jian Duan, Jinlin Xiao, Quan Ren, Pengfei Zhou, Jian Yan, Youping Li, Xiaoxing Xiong, Erming Zeng

**Affiliations:** ^1^Department of Neurosurgery, The First Affiliated Hospital of Nanchang University, Nanchang, China; ^2^Department of Neurosurgery, Renmin Hospital of Wuhan University, Wuhan, China

**Keywords:** moyamoya disease, apolipoprotein E, biomarkers, cholesterol, carotid artery

## Abstract

**Objective:**

The mechanisms underpinning Moyamoya disease (MMD) remain unclear, and effective biomarkers remain unknown. The purpose of this study was to identify novel serum biomarkers of MMD.

**Methods:**

Serum samples were collected from 23 patients with MMD and 30 healthy controls (HCs). Serum proteins were identified using tandem tandem-mass-tag (TMT) labeling combined with liquid chromatography-tandem mass spectrometry (LC-MS/MS). Differentially expressed proteins (DEPs) in the serum samples were identified using the SwissProt database. The DEPs were assessed using the Kyoto Encyclopedia of Genes and Genomes (KEGG) database, Gene Ontology (GO), and protein-protein interaction (PPI) networks, and hub genes were identified and visualized using Cytoscape software. Microarray datasets GSE157628, GSE189993, and GSE100488 from the Gene Expression Omnibus (GEO) database were collected. Differentially expressed genes (DEGs) and differentially expressed miRNAs (DE-miRNAs) were identified, and miRNA targets of DEGs were predicted using the miRWalk3.0 database. Serum apolipoprotein E (APOE) levels were compared in 33 MMD patients and 28 Moyamoya syndrome (MMS) patients to investigate the potential of APOE to be as an MMD biomarker.

**Results:**

We identified 85 DEPs, of which 34 were up- and 51 down-regulated. Bioinformatics analysis showed that some DEPs were significantly enriched in cholesterol metabolism. A total of 1105 DEGs were identified in the GSE157628 dataset (842 up- and 263 down-regulated), whereas 1290 were identified in the GSE189993 dataset (200 up- and 1,090 down-regulated). The APOE only overlaps with the upregulated gene expression in Proteomic Profiling and in GEO databases. Functional enrichment analysis demonstrated that APOE was associated with cholesterol metabolism. Moreover, 149 miRNAs of APOE were predicted in the miRWalk3.0 database, and hsa-miR-718 was the only DE-miRNA overlap identified in MMD samples. Serum APOE levels were significantly higher in patients with MMD than in those without. The performance of APOE as an individual biomarker to diagnose MMD was remarkable.

**Conclusions:**

We present the first description of the protein profile of patients with MMD. APOE was identified as a potential biomarker for MMD. Cholesterol metabolism was found to potentially be related to MMD, which may provide helpful diagnostic and therapeutic insights for MMD.

## 1. Introduction

Moyamoya disease (MMD) is a chronic, rare cerebrovascular disease characterized by progressive vascular occlusion affecting the internal carotid arteries (ICAs) and formation of a compensatory network of fragile vessels at the base of the brain (1, 2). MMD is found globally, especially in East Asian countries, such as Japan, China, and Korea (1, 3). In China, the prevalence of the disease in Nanjing is 3.92/100,000 (4, 5). MMD can cause ischemic or hemorrhagic stroke, and bleeding is the main cause of death in adults with moyamoya disease (1). There is a lack of effective drugs to treat moyamoya disease as the exact mechanism of MMD pathogenesis remains unclear.

Recently, an increasing number of studies have indicated the association of moyamoya disease with RNF213 variant, a key antimicrobial protein that strengthens the role of infectious or autoimmune stimuli as a contributing factor to MMD onset (2, 6). However, not all patients with MMD have the RNF213 variant, indicating that the pathology of MMD is complex, including genetic and environmental factors, and innate angiogenetic capacity (7).

Many studies have shown IgG, IgM, and C3 are found on the vascular wall patients with MMD. Moreover, the inflammatory response causes hyperplasia of intimal vascular smooth muscle cells and neovascularization through the proliferation of endothelial cells, resulting in lumen stenosis and reformation of collateral circulation (8, 9). Circulating proteins such as MMP-9 and caveolin-1 can help regulate the extracellular matrix of the vessel wall, resulting in pathological neovascularization with defective vessel structure, inducing negative arterial remodeling and impairing angiogenesis in MMD (10, 11). Therefore, it is important to detect serum proteins in patients with MMD to further understand the pathogenesis of MMD.

To explore whether there is a serum biomarker for moyamoya disease, we used proteomics to analyze the differentially expressed proteins between moyamoya disease and healthy people. We searched for overlaps with differentially expressed genes in vascular tissues of moyamoya disease in the GEO database. Bioinformatic analysis of the differentially expressed proteins was further verified using independent samples. In summary, we identified a novel serum biomarker and proposed a potential pathogenic mechanism for the development of MMD.

## 2. Materials and methods

### 2.1. Patients and serum samples

This study analyzed 114 serum samples obtained from 56 patients diagnosed with MMD, 28 Moyamoya syndrome (MMS) patients, and 30 healthy controls at the First Affiliated Hospital of Nanchang University (Nanchang, China) between September 2019 and August 2021. The study protocol was conducted in accordance with the Declaration of Helsinki and was approved by the Research Ethics Committee of the First Affiliated Hospital of Nanchang University (Nanchang, China). All participants voluntarily signed the informed consent forms.

MMD was diagnosed according to guidelines proposed by the Ministry of Health and Welfare of Japan. The diagnostic criteria are shown in Supplementary material.

### 2.2. TMT-based quantitative serum proteomics

In the MMD group, three pooled samples were generated by random mixing of three or four samples, and three pooled samples were generated by random mixing of every 10 samples in the HCs group. All pooled samples were lysed, trypsin-digested, and analyzed using the Tandem Mass Tag-labeled serum proteome. TMT analysis was performed according to a previously reported method (12, 13). Clinical and group information is shown in Supplementary Datasheet 1.

### 2.3. Differential expression analyses of serum proteomics

To reliably identify differential proteins, we applied fold change of proteins ≥ 1.2 and *p*-value < 0.05 to screen and filter the identified proteins using the “limma” R package (14).

### 2.4. Microarray datasets of MMD and preprocessing

We used “moyamoya disease” as a keyword on the Gene Expression Omnibus (GEO) database (https://www.ncbi.nlm.nih.gov/geo/), mRNA microarray datasets were obtained with the accession no. GSE189993 and GSE157628 contained middle cerebral artery (MCA) vascular wall tissue data from 32 MCA with MMD samples and 20 control samples (12 patients with internal carotid artery aneurysm and eight epilepsy patients).

miRNA microarray data were also collected from the GEO database under Accession No. GSE100488, which analyzed the circulating miRNA profiles from 10 peripheral blood plasma samples with MMD and 10 peripheral blood plasma samples from healthy controls.

### 2.5. Microarray datasets analysis

All the samples were normalized using the “limma” R package. In this study, the GEO2R platform (http://www.ncbi.nlm.nih.gov/geo/geo2r/) was used to detect DEGs and DE-miRNA between MMD and control groups. The truncation criteria for DEGs were set with | log 2 fold change | > 1.5, *P* < 0.05, and DE-miRNAs were set with | log 2 fold change | > 0.5, *P* < 0.05.

### 2.6. Functional and pathway enrichment analysis

The functional enrichment analysis of DEPs and DEGs were performed on the Gene ontology (GO), and Kyoto Encyclopedia of Genes and Genomes (KEGG) using “enrichGO” and “enrichKEGG” R package.

### 2.7. Protein-protein interaction network analysis

The list of DEPs and DEGs was updated to the STRING database (version 11.5; https://cn.string-db.org/) to construct protein–protein interaction (PPI) networks, with the minimum required interaction score was set to 0.4. Cytoscape software was used to obtain the hub genes and visualize the PPI network map.

### 2.8. Target DE-miRNAs prediction of gene

The miRWalk database (Version 3.0; http://mirwalk.umm.uni-heidelberg.de/) was used to predict the DE-miRNA targets of mRNA.

### 2.9. Enzyme-linked-immunosorbent serologic assay validation assay

The serum samples were diluted 1:32,000 in the kit-supplied assay buffer. Next, 20 μL of standards was added to duplicates in a clear, 96-well half-area plate (Costar Corporation, USA). Serum concentrations were assessed using a highly sensitive enzyme-linked immunosorbent assay kit. The ELISA assay was performed according to the manufacturer's instructions. The absorbance was read at 560 nm in a Multiskan GO microplate spectrophotometer (Thermo Fisher Scientific), and the results were acquired by interpolation from a 4-parametric logistic curve generated by Thermo Scientific SkanIT Software version 3.2.

### 2.10. Statistical analysis

Continuous variables are presented as mean ± standard deviation (SD). Categorical data were reported as counts and proportions in each group. The data between the groups were compared using the chi-square test (Fisher's exact test, where appropriate) for categorical variables or 2-tailed Student *t*-test (Mann-Whitney *U*-test, where appropriate) for continuous variables. Statistical significance of all data was indicated by *P* < 0.05. A heatmap was plotted using Sangerbox (Version 3.0; http://vip.sangerbox.com), an online platform for data analysis and visualization.

### 2.11. ROC analysis

We applied the receiver operating characteristic (ROC) curve and used the area under the curve (AUC) to evaluate diagnostic accuracy. The R package “pROC” (version 1.17.0.1) was used to analyze the results and visualize the data.

## 3. Results

### 3.1. Screening of differentially expressed proteins

**Figure 1:**
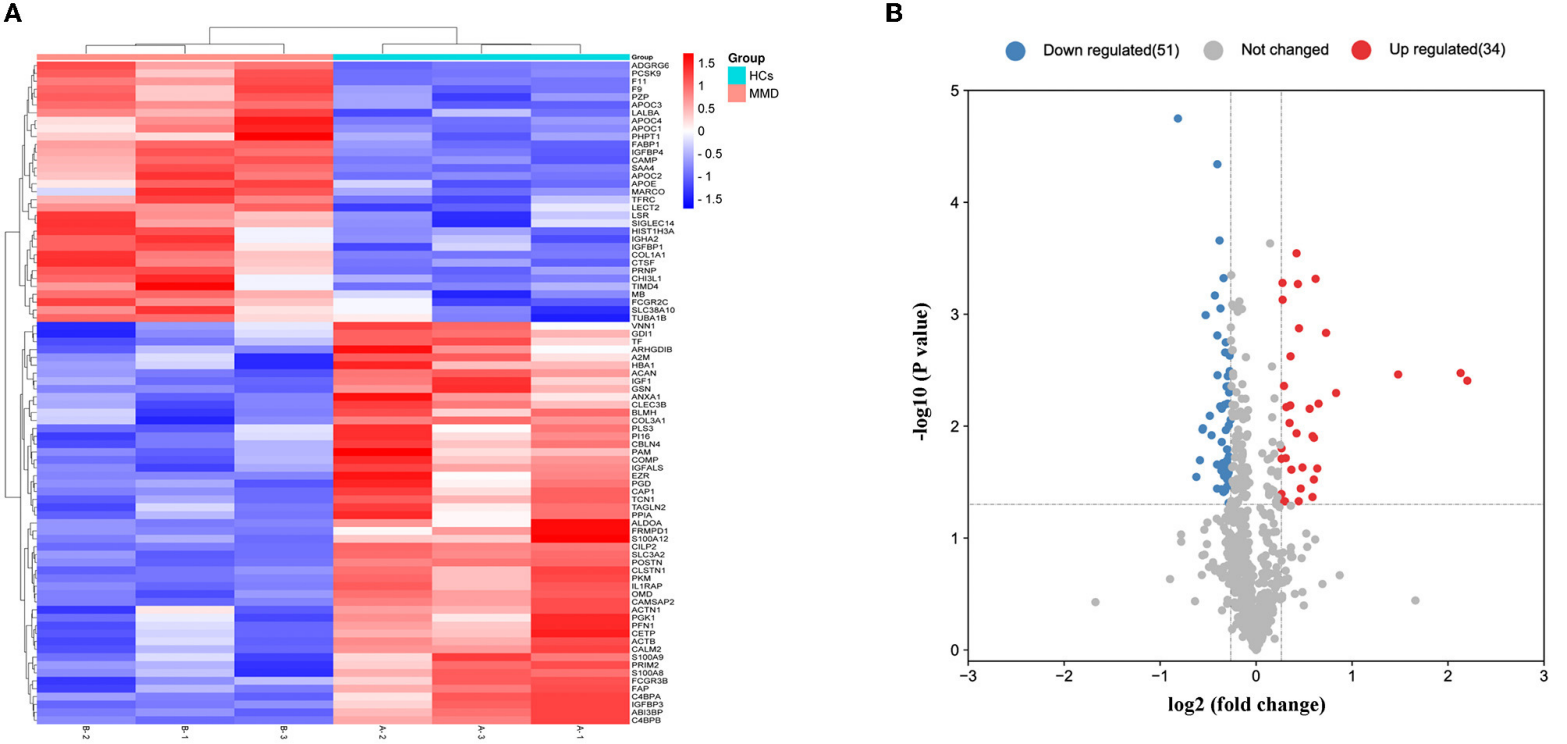
Detection of differentially expressed proteins. Overall distribution of differentially expressed proteins. **(A)** Heat map of 85 differentially expressed proteins in the two groups. In the color bar, red represents high expression, and purple represents low expression. **(B)** Volcano plot of the differentially expressed proteins identified in the two groups.

Comparative proteomic analysis of serum from 23 patients with Moyamoya disease (MMD) and 30 healthy controls was performed using TMT labeling following LC–MS/MS analysis. The clinical information is shown in Supplementary Table 1. A total of 705 proteins with unique peptides were identified (Supplementary Table 2). The differentially expressed proteins between every two groups were obtained according to the criteria of (fold-change ≥ 1.2 and *P* < 0.05). The results showed that 85 DEPs were upregulated, 34 proteins were significantly upregulated, and 51 were significantly downregulated in MMD compared with HCs (Figures 1A, B).

### 3.2. Bioinformatics analysis of differentially expressed proteins

**Figure 2:**
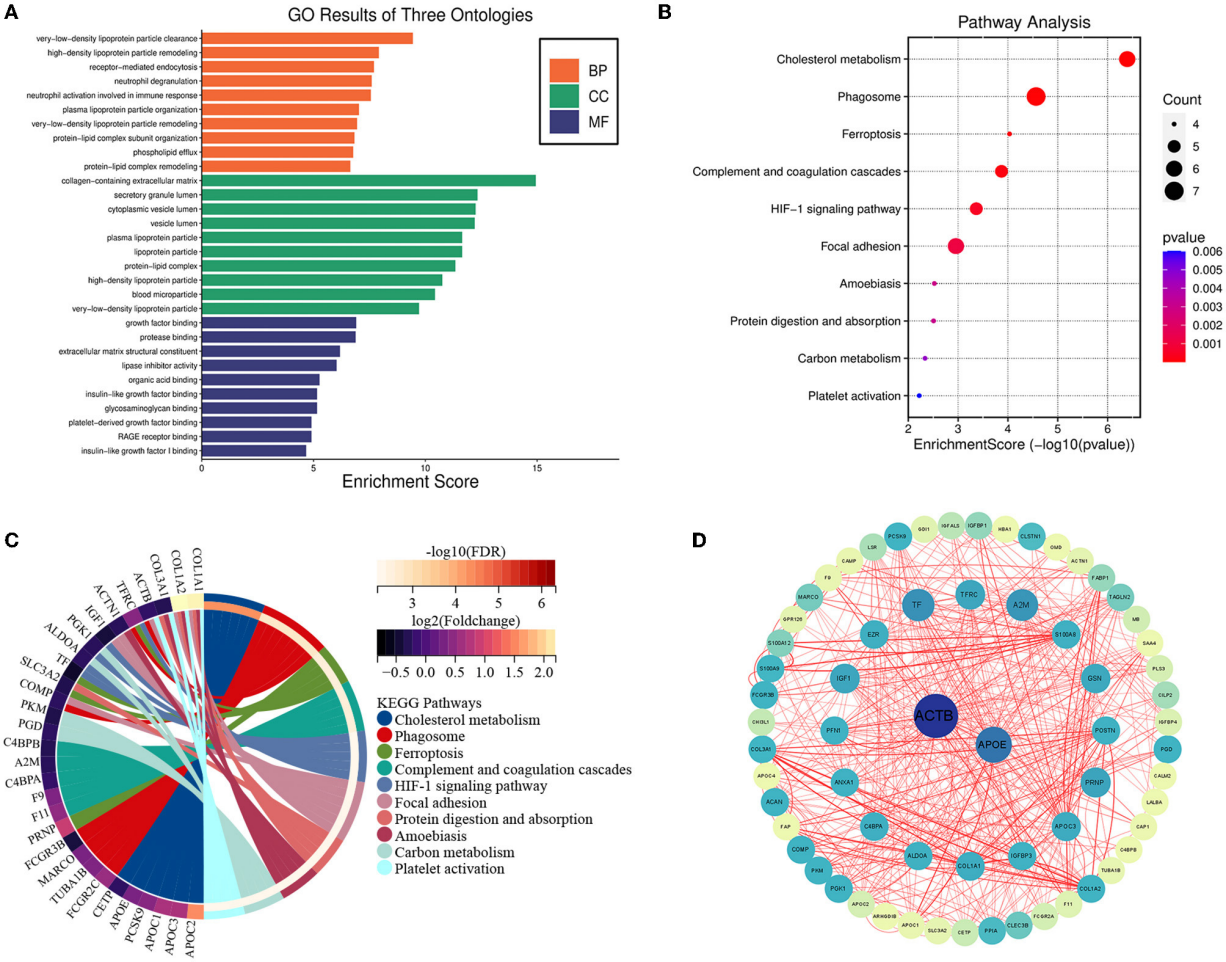
Bioinformatics analysis of differentially expressed proteins. Functional enrichment and protein–protein interaction analysis **(A)** GO enrichment results for DEPs in biological processes. GO, Gene Ontology; BP, biological processes; CC, cellular components; MF, molecular function. **(B)** Bubble chart displaying the enrichment of differentially expressed genes and the top 10 enriched KEGG pathways. **(C)** Chord plot displaying the enriched enrichment network of differentially expressed genes and the top 10 enriched KEGG pathways. **(D)** The protein-protein interaction network was analyzed using the STRING database. There were two nodes and 83 edges in the network.

GO enrichment analysis revealed that these DEPs were enriched in three GO terms: biological process (BP), cellular component (CC), and molecular function (MF). BP processes include very-low-density lipoprotein particle clearance, high-density lipoprotein particle remodeling, and receptor-mediated endocytosis. The CC included the collagen-containing extracellular matrix, secretory granule lumen, cytoplasmic vesicle lumen, high-density lipoprotein particle and very-low-density lipoprotein particle. With respect to MF, DEPs were primarily enriched in growth factor binding, protease binding, extracellular matrix and lipase inhibitor activing (Figure 2A). The results showed that these genes were functionally associated with cholesterol metabolism.

These DEPs were also enriched in KEGG pathways, including the cholesterol metabolism, Phagosome, Ferroptosis pathways, complement and coagulation cascades, HIF-1 signaling pathway, and Focal adhesion (Figures 2B, C).

To further investigate the links of the 85 DEPs, the online STRING database was used to analyze and construct a Protein-Protein Interaction (PPI) network. We identified two hub genes associated with MMD according to the results of the PPI analysis, including ACTB and APOE, and the results were visualized using Cytoscape software (Figure 2D).

### 3.3. Overlaps gene between with proteomic profiling and GEO database and bioinformatics analysis

**Figure 3:**
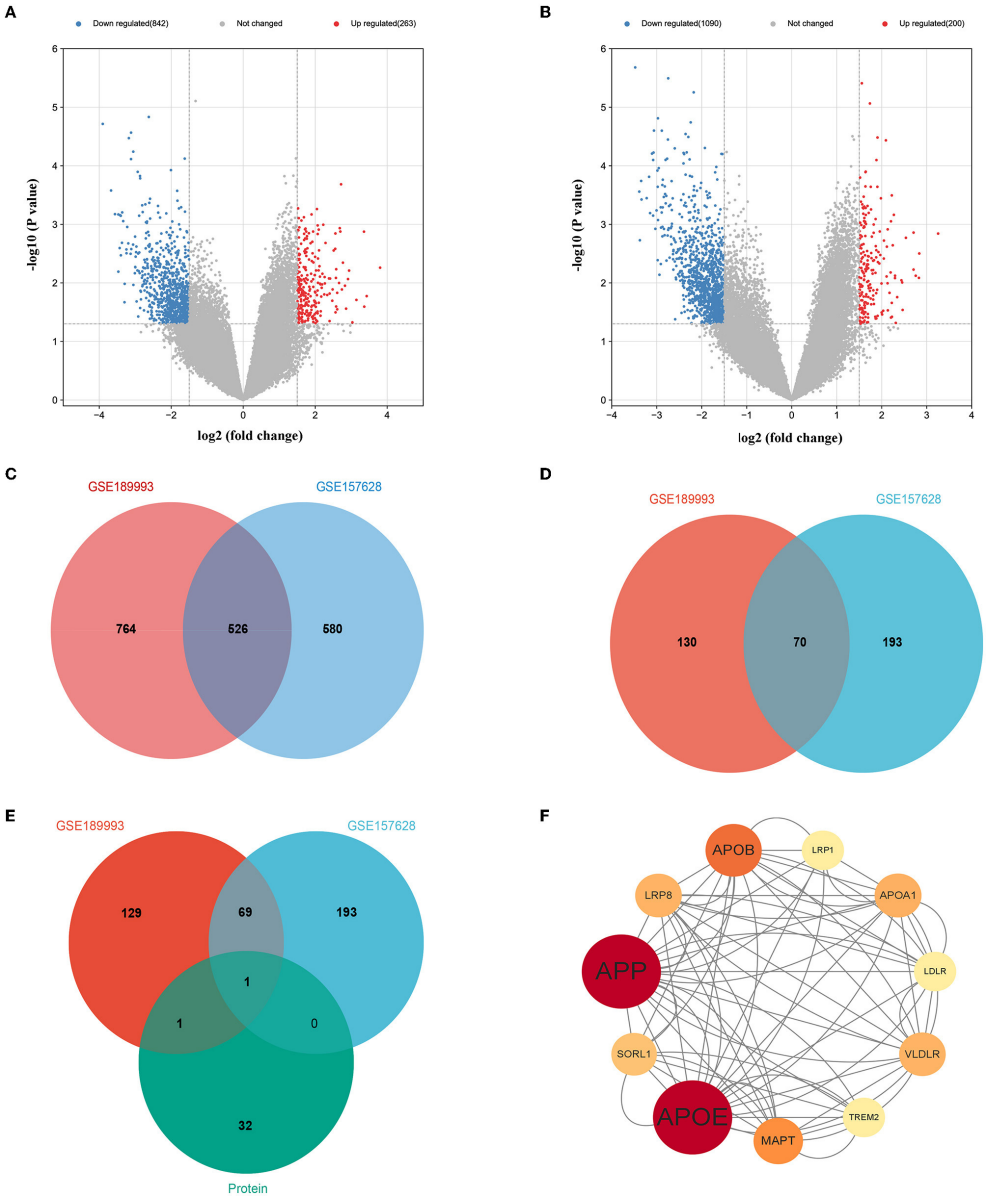
Overlaps gene between with proteomic profiling and GEO database and bioinformatics analysis. **(A)** Volcano plot of differentially expressed genes in the GSE157628 dataset. **(B)** Volcano plot of differentially expressed genes in the GSE189993 dataset. **(C)** Venn diagram showing the intersection of differentially expressed genes between the GSE189993 and GSE157628 datasets. **(D)** Venn diagram showing the intersection of highly expressed genes between GSE189993 and GSE157628 datasets. **(E)** Venn diagram showing the intersection of highly expressed genes in the GEO database and Proteomic Profiling. **(F)** The protein-protein interaction network of APOE was constructed using Cytoscape.

In our study, we obtained two mRNA microarray datasets from the GEO (GSE157628 and GSE189993) and searched for DEGs using GEO2R. A total of 263 down- and 842 up-regulated DEGs were identified from the GSE157628 dataset, and 1,090 down- and 200 up-regulated DEGs were identified from the GSE189993 dataset. A volcano plot of each gene expression profile was prepared (Figures 3A, B). Venn diagram analysis revealed that 526 DEGs and 70 down-regulated overlapping DEGs were found in MMD compared to the control (Figures 3C, D). In addition, APOE only overlapped with up-regulated gene expression in proteomic profiling and in the GEO database (Figure 3E). Further functional enrichment analysis suggested that APOE is associated with cholesterol metabolism (Figure 3F).

### 3.4. Identification of DE-miRNAs between MMD and MCA

**Figure 4:**
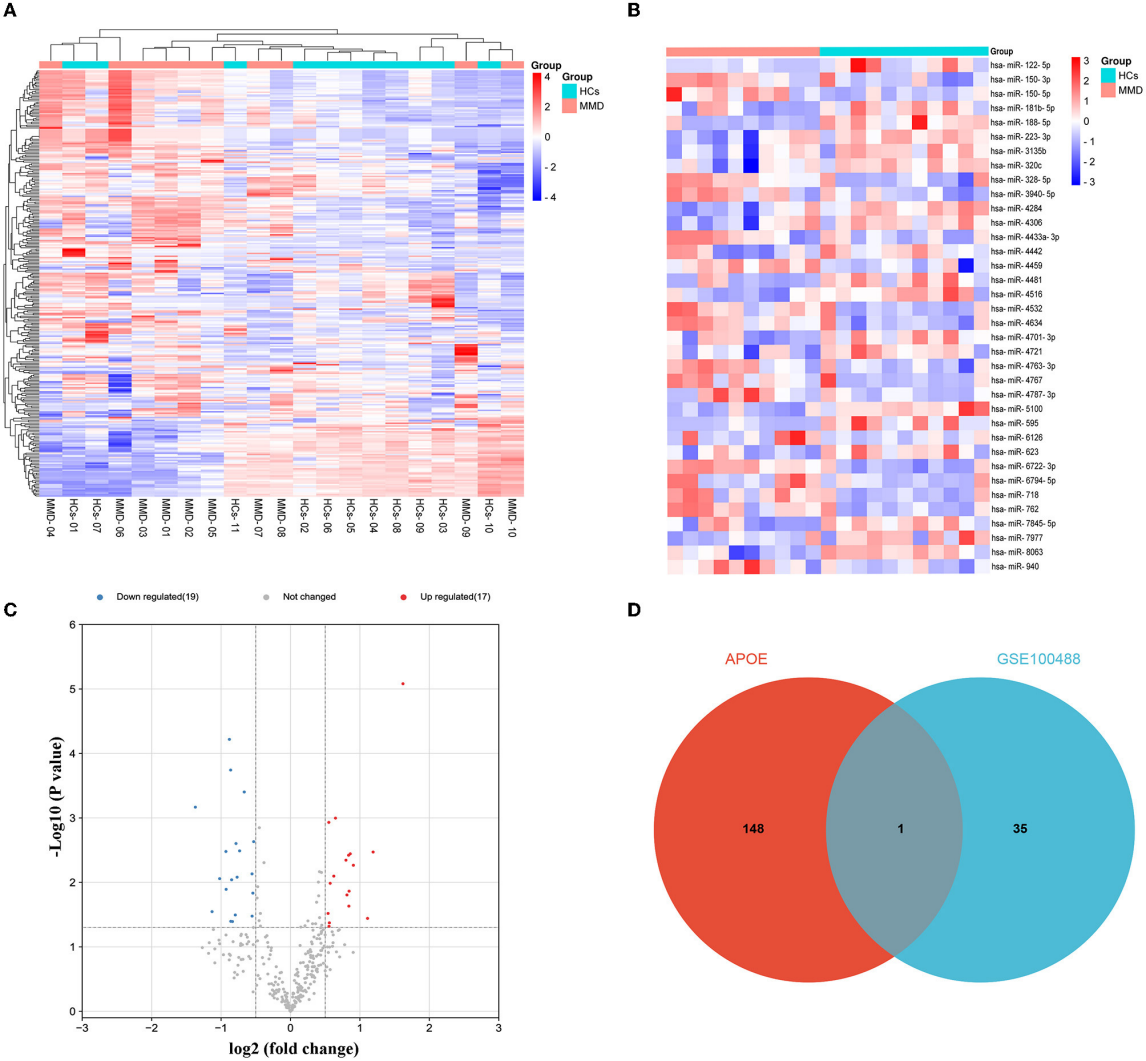
Identification of DE-miRNAs between MMD and MCA. Differentially expressed miRNAs were identified using the GSE100488 dataset. **(A)** Heat map of miRNAs in the GSE100488 dataset (red indicates high expression, and blue indicates low expression). **(B)** Heat map of the 36 differentially expressed miRNAs in the GSE100488 dataset (red indicates high expression and blue indicates low expression). **(C)** Volcano plots of differentially expressed miRNAs in the two groups. **(D)** The Venn diagram reveals the intersection of DE-miRNAs between the GSE100488 dataset and predicted miRNAs of APOE using the miRWalk3 database.

Potential upstream miRNAs of mRNAs were predicted by miRWalk3 database, and the intersection with DE-miRNA (hsa-miR-718) in the GSE100488 dataset was used to obtain candidate miRNAs of APOE. The heat map shows the total number of miRNAs and DE-miRNAs in the GSE100488 dataset (Figures 4A, B). Thirty-six DE-miRNAs were identified in the peripheral blood plasma of MMD patients and normal healthy controls, including 19 down- and 17 up-regulated DE-miRNAs (Figure 4C). Venn diagram analysis revealed that there was only one shared DE-miRNA (hsa-miR-718) in the GSE100488 dataset and predicted miRNAs of APOE using the miRWalk3 database (Supplementary Table 3; Figure 4D).

### 3.5. Validation of APOE as an individual biomarker in an independent cohort

**Figure 5:**
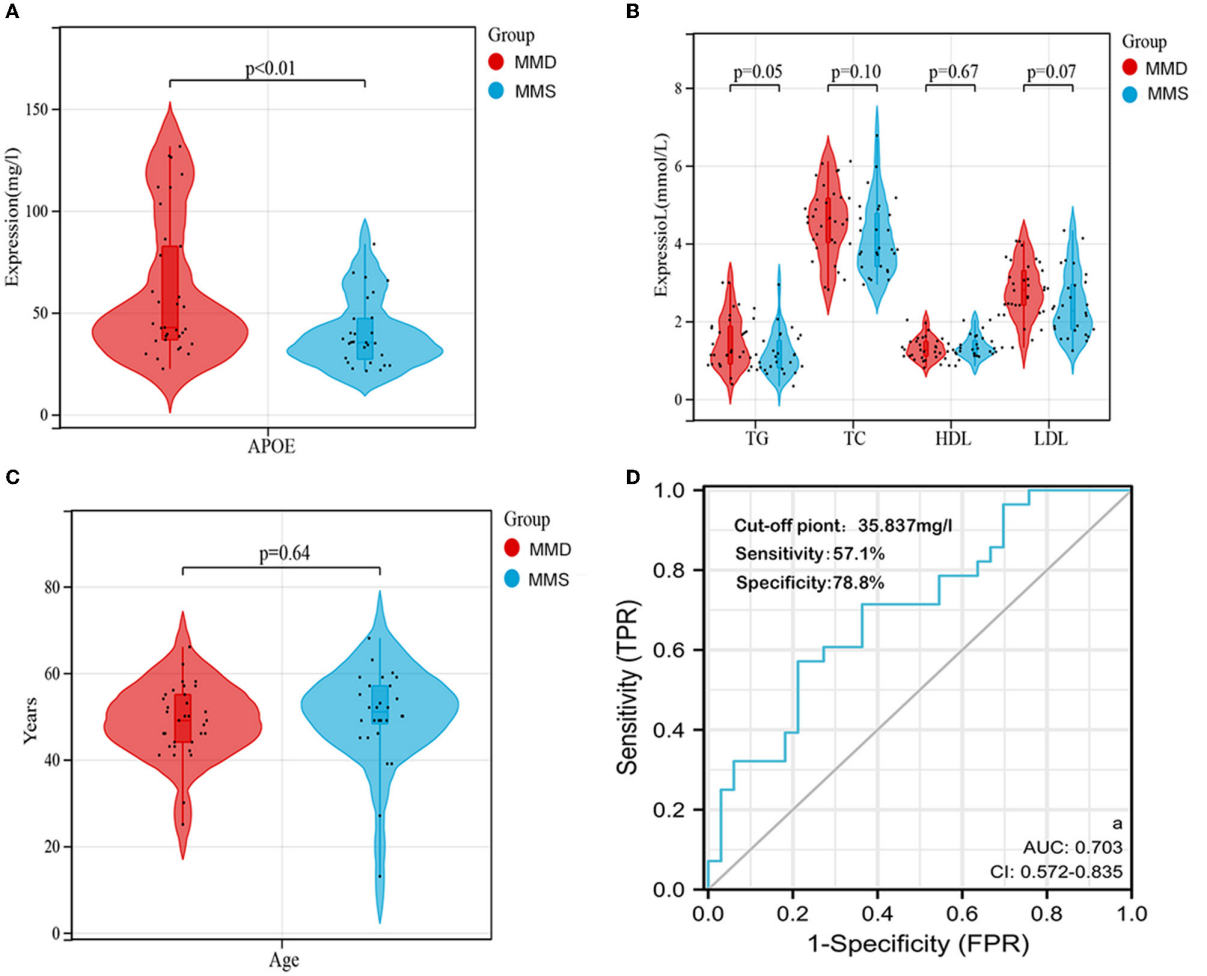
Validation of APOE as an individual biomarker in an independent cohort. **(A)** Serum APOE expression levels in the two groups were visualized using violin plots. **(B)** Serum TG, TC, LDL, and HDL levels between the two groups are shown by violin plots. **(C)** Distribution of age between the two groups is shown using violin plots. **(D)** ROC curve and corresponding AUC of APOE in the independent validation cohort (*n* = 61). DE, differentially expressed; miRNA, microRNA.

We collected 33 patients with MMD and 28 patients without MMD to investigate the potential of APOE as a biomarker for moyamoya disease. The clinical information is shown in Supplementary Table 4. The levels of APOE protein in serum samples from MMD and MMS patients were further validated using ELISA (Supplementary Table 5). As shown in Table 1, we found that the serum levels of APOE protein differed significantly between the two groups (*P* < 0.001; Figure 5A). The expression levels of triglyceride (TG), total cholesterol (TC), low-density lipoprotein (LDL), and high-density lipoprotein (HDL) in MMD serum samples, while age and sex showed no significant differences compared to the control group (*P* > 0.05; Figures 5B, C; Table 1).

**Table 1:** Comparison of the baseline characteristics of patients with MMD and MMS.

**Variable**	**MMD (mean ±SD), *n* = 33**	**MMS (mean ±SD), *n* = 28**	***P*-value**
Sex, female	21 (63.6%)	14 (50%)	0.29
Age (years)	48.76 ± 8.41	49.96 ± 10.90	0.64
TG (mmol/L)	1.47 ± 0.67	1.16 ± 0.54	0.05
TC (mmol/L)	4.56 ± 0.91	4.16 ± 0.95	0.1
HDL (mmol/L)	1.31 ± 0.29	1.34 ± 0.30	0.67
LDL (mmol/L)	2.79 ± 0.71	2.42 ± 0.83	0.07
APOE (mg/l)	60.75 ± 33.99	39.93 ± 16.54	0.0003

P < 0.05 was considered statistically significant.

To determine whether APOE has diagnostic significance in MMD patients, ROC analyses were conducted to explore the sensitivity and specificity of DEGs for MMD diagnosis. The results showed that APOE has diagnostic value in differentiating patients with MMD from MMS patients (Figure 5D). The AUC value of serum APOE was 0.703 (95% CI, 0.572–0.835), and the cut-off point was 35.837 mg/l (57.1% sensitivity and 78.8% specificity).

## 4. Discussion

MMD is typically characterized by progressive narrowing or stenosis, but its etiology and pathogenesis remain unclear (7). Previous studies have confirmed that multiple molecular pathways are involved in the pathophysiological processes of MMD, including smooth muscle cell and extracellular matrix proliferation, intima concentric fibrocellular hyperplasia, extracellular interstitial remodeling, apoptosis, and vascular inflammation (15–20). In addition, the investigation of MMD has been limited by the difficulty of sample collection and lack of *in vitro* and *in vivo* models. Proteins are direct effectors of biological mechanisms, and serum proteins are widely used as biomarkers for clinical diagnosis and mechanistic research of many diseases (21). In this study, we compared serum protein profiles of patients with MMD and healthy individuals to identify candidate biomarkers, then a potential pathogenic mechanism of MMD was proposed by bioinformatics. Furthermore, the serum biomarker for enzyme-linked immunosorbent assay (ELISA) was validated in independent cohorts.

Eighty-five DEPs were identified and GO term analysis indicated that these genes are significantly associated with cholesterol metabolism. APOE was the most significant genes among these DEGs, which were identified as hub genes by PPI network analysis. Besides, through KEGG pathway analysis, we found that these DEPs were mainly enriched in cholesterol metabolism, and may be involved in Ferroptosis pathways, HIF-1 signaling pathway and immune system. Taking the intersection of up-regulated DEPs and two GEO datasets of up-regulated DEGs, only APOE was identified. In the GSE188993 and GSE157628 datasets, middle cerebral artery microsamples of patients with MMD and control groups were detected by microarray assay, and high expression of APOE was found in two databases (22, 23). However, the role of APOE in MMD has remained unexplored.

The APOE gene is located on the long arm of chromosome 19. It is polymorphic, with three major alleles (ε2, ε3, and ε4) that significantly alter the structure and function of APOE (24). Previous studies have shown that APOE gene polymorphisms may play a role in micro-bleeds in patients with MMD, but the mechanism is unclear (25). Baitsch et al. found that APOE is derived from endothelium-resident macrophages, which can cause vascular remodeling by influencing nitric oxide soluble (NOS) factors (26). Some studies have shown that macrophage infiltration has also been observed in MMD, and the NOS guanylate cyclase-cyclic guanosine monophosphates (NO-GC-cGMP) signaling pathways are associated with vascular remodeling in MMD (27, 28). Therefore, to further determine the relationship between APOE and the occurrence and development of MMD, through a combination of GO enrichment and KEGG pathway enrichment analyses, we identified APOE as a key regulatory protein associated with cholesterol metabolism. Previous studies have also shown that APOE is involved in cholesterol metabolism (29–32). Therefore, we speculated that cholesterol metabolism may have implications for MMD. We also found that hsa-miR-718 was the only DE-miRNA to overlap in the GEO dataset and predicted APOE miRNAs. Hsa-miR-718 has not been previously reported to regulate APOE, which requires further experimental verification.

Additionally, the serum proteins of APOE were further validated by ELISA in serum samples from patients with and MMS patients. We found that the serum concentration of APOE protein differed significantly between MMD and MMS patients (*P* < 0.001). Identifying specific protein markers is crucial for improving MMD diagnosis. Our study showed that APOE has diagnostic value with an AUC value of 0.703.

Nevertheless, the present study has some limitations. First, the serum sample size was small, which can lead to statistical biases in the analyses performed to investigate diagnostic value. Furthermore, although the results of this study preliminarily suggest an association between APOE and hsa-miR-718, well designed *in vitro* and *in vivo* experiments are required to confirm this result. Finally, the role of APOE in MMD and the regulation of cholesterol metabolism remains unclear, and further research is needed to confirm its function and molecular mechanism.

Collectively, our findings suggest that serum APOE is a potential biomarker in patients with MMD, and cholesterol metabolism may be involved in MMD.

## 5. Conclusion

Using TMT-labeling HPLC-MS quantitative proteomics technology, we screened and identified biomarkers of MMD and analyzed them at the serum level. Specific serum APOE proteins for MMD were selected and evaluated to determine their feasibility as candidate diagnostic markers of MMD. The present study found that cholesterol metabolism might be involved in the development of MMD. These findings may provide important clues for further studies to clarify the pathophysiology of MMD.

## Data availability statement

The original contributions presented in the study are publicly available. This data can be found here: ProteomeXchange, https://www.proteomexchange.org/, PXD039975.

## Ethics statement

The studies involving human participants were reviewed and approved by the Research Ethics Committee of the First Affiliated Hospital of Nanchang University. The patients/participants provided their written informed consent to participate in this study.

## Author contributions

HW, JXu, JS, and EZ conceived and designed the study. HW and JS analyzed and interpreted the data. JD, JXi, QR, PZ, JY, XX, and YL participated in the sample collection and data acquisition. All authors participated in drafting the manuscript, read and approved the final version of the manuscript, and provided consent for publication.
