# Supplementary material for: APOE as potential biomarkers of moyamoya disease
Source: Front Neurol. 2023 May 9;14:1156894. doi: 10.3389/fneur.2023.1156894 (PMC10203507; doi:10.3389/fneur.2023.1156894)
Supplement: Supplementary file 6 [file Data_Sheet_1.docx]

The diagnostic of MMD criteria were as follows: 1) stenosis or occlusion around the terminal portion of the internal carotid arteries, 2) moyamoya vessels at the brain base, and 3) exclusion of diseases with similar angiographic characteristics (e.g., arteriosclerosis, autoimmune disease, meningitis, brain neoplasm, Down syndrome, neurofibromatosis type 1, head trauma, or irradiation of the head). Moyamoya syndrome (MMS) is refer to the moyamoya-like vasculopathy with associated risk factors, such as neurofibromatosis type, down syndrome, thyroid disease, cranial irradiation, sickle cell anemia, and so on.
